# Increased Fracture Collapse after Intertrochanteric Fractures Treated by the Dynamic Hip Screw Adversely Affects Walking Ability but Not Survival

**DOI:** 10.1155/2016/4175092

**Published:** 2016-02-03

**Authors:** Christian Fang, Paata Gudushauri, Tak-Man Wong, Tak-Wing Lau, Terence Pun, Frankie Leung

**Affiliations:** ^1^Department of Orthopaedics and Traumatology, Queen Mary Hospital, The University of Hong Kong, Hong Kong; ^2^Tbilisi State Medical University, Tbilisi, Georgia

## Abstract

In osteoporotic hip fractures, fracture collapse is deliberately allowed by commonly used implants to improve dynamic contact and healing. The muscle lever arm is, however, compromised by shortening. We evaluated a cohort of 361 patients with AO/OTA 31.A1 or 31.A2 intertrochanteric fracture treated by the dynamic hip screw (DHS) who had a minimal follow-up of 3 months and an average follow-up of 14.6 months and long term survival data. The amount of fracture collapse and shortening due to sliding of the DHS was determined at the latest follow-up and graded as minimal (<1 cm), moderate (1-2 cm), or severe (>2 cm). With increased severity of collapse, more patients were unable to maintain their premorbid walking function (minimal collapse = 34.2%, moderate = 33.3%, severe = 62.8%, and *p* = 0.028). Based on ordinal regression of risk factors, increased fracture collapse was significantly and independently related to increasing age (*p* = 0.037), female sex (*p* = 0.024), A2 fracture class (*p* = 0.010), increased operative duration (*p* = 0.011), poor reduction quality (*p* = 0.000), and suboptimal tip-apex distance of >25 mm (*p* = 0.050). Patients who had better outcome in terms of walking function were independently predicted by younger age (*p* = 0.036), higher MMSE marks (*p* = 0.000), higher MBI marks (*p* = 0.010), better premorbid walking status (*p* = 0.000), less fracture collapse (*p* = 0.011), and optimal lag screw position in centre-centre or centre-inferior position (*p* = 0.020). According to Kaplan-Meier analysis, fracture collapse had no association with mortality from 2.4 to 7.6 years after surgery. In conclusion, increased fracture collapse after fixation of geriatric intertrochanteric fractures adversely affected walking but not survival.

## 1. Introduction

Implants designed for fixation of osteoporotic hip fracture typically allow controlled sliding and collapse to improve bony contact and healing [1]. The dynamic hip screw (DHS) is one of the most widely used and successful implants for the treatment of stable intertrochanteric fractures with such design concept [2, 3]. Conversely, statically locked implants that aim at static fixation have yielded unacceptably high rate of failures for routine osteoporotic hip fractures because of the lack of dynamic compression and a lack of technical tolerance [4, 5]. Cephalomedullary fixation devices [6] and DHS with trochanter stabilizing plate [7] are effective means in preventing excessive collapse in unstable fractures (such as the AO/OTA type 31.A3 [8, 9]) but still much debated for routing use in more stable and intermediate patterns (such as types 31.A1 and A2).

Fracture collapse is sometimes associated with fixation failure [10] and believed to impair the hip abduction lever arm [11]. Researchers have pointed out that, for intracapsular hip fractures, increased fracture collapse accounted for poorer functional recovery [12, 13]. In young patients with trochanteric fractures, previous studies have shown that significant shortening of more than 2 cm is associated with impaired functional outcome [14] and previous studies have shown that DHS are associated with more pronounced shortening than cephalomedullary devices in young bone [14]. Currently, it has not been clearly studied whether collapse also adversely affects functional and survival outcomes for elderlies with trochanteric hip fractures.

Our objective was to test the hypothesis on whether patients with increasing degree of fracture collapse and shortening after DHS had impaired walking and increased incidence of adverse events. We carried out a secondary analysis of a consecutive prospective cohort of patients with trochanteric fractures treated with two similar DHS designs [15].

## 2. Patients and Methods

We reviewed a consecutive cohort of 433 patients with AO/OTA [8, 9] 31.A1 and 31.A2 fractures operated from 2007 to 2012 with DHS lag screws or DHS blades (both from formerly Synthes, Oberdorf, Switzerland). Only the cephalic portion of the implant differed and both were mounted on a standard nonlocked four-hole DHS slide plate. All included patients were over 50 years and patients with pathological fractures were excluded. Our hip fracture pathway [16] mandated surgical treatment as soon as possible unless the patient was medically unfit. A standard lateral subvastus approach was used for all patients on a traction table by closed or optional open assisted reduction. Patients followed a multidisciplinary rehabilitation protocol and were allowed immediate full weight bearing exercise. Upon follow-up, the functional status in walking and radiographs were analysed for collapse and complications.

**Figure 1:**
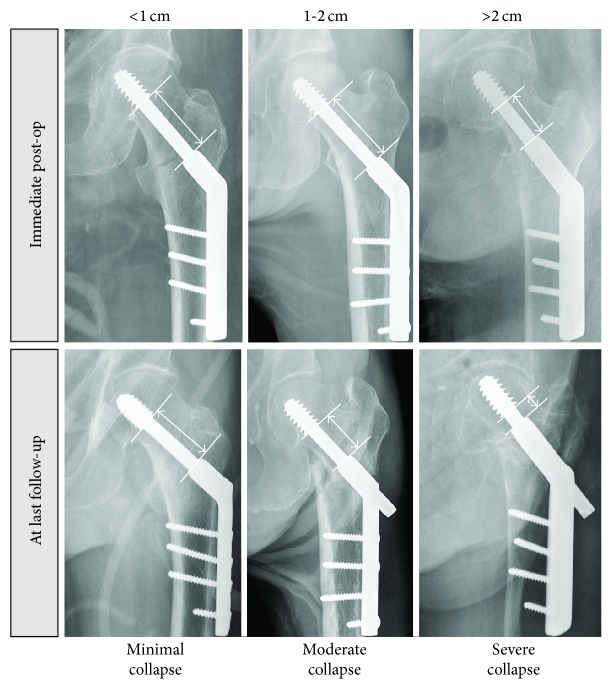
Grading of severity of fracture collapse and shortening.

The degree of fracture collapse was determined by comparing the intraoperative and the latest anteroposterior radiograph. The remaining length of the lag screw or blade available for collapse was noted. The amount of collapse was determined at fracture union or at time of latest radiograph before any catastrophic mechanical failure. The degree of collapse was graded as minimal if this was not detectable when comparing the two radiographs or less than 1 cm, moderate if this was from 1 to 2 cm, and severe if this was more than 2 cm. The core diameter of the lag or the length of the side-plate barrel is used as reference dimensions to correct for the effects of magnification and rotation (see Figure 1).

Perioperative patient, fracture, and surgical variables were compared for factors associated with different degree of collapse. These included age, gender, American Anesthesia Society (ASA) score, hours from admission to surgery (more than 48 hours versus less than 48 hours), Mini Mental State Exam (MMSE) score [17], Modified Barthel Index [18] (MBI), surgeon experience (specialists with more than six years of experience versus trainees with less), and premorbid walking status. Walking status was graded as independent walker for those who did not require any assistance; assisted walkers if some degree of assistance was needed for activity of daily living; and nonfunctional walkers either if considerable assistance is needed or if the patient cannot walk at all. Implant position was defined as optimal if the fixation device was placed at the centre-centre or centre-inferior position of the femoral head; the tip-apex distance, as defined by Baumgaertner et al. [19], is optimal when less than 25 mm. Fracture reduction was defined according to Baumgaertner et al. [20] depending on the presence of a translation of more than 4 mm or varus of more than 5 degrees (good or acceptable reduction) or both (poor reduction).

The primary outcome was the best walking status achieved after rehabilitation using the same definition as the premorbid walking status. The premorbid walking grade was compared with postoperatively to determine whether there was a deterioration by one grade or more. The secondary outcomes were mortality, presence of complications including lateral wall fractures, implant migration in femoral head, cutout, side-plate pullout, nonunion, infection, and reoperations. Mortality data is recovered from the local territory wide electronic death registry, while survival is determined according to any patient attendance documented in the electronic public health care system which offers more than 90% territorial coverage.

SPSS software version 23 (IBM, Armonk NY, USA) was used for statistical analysis. The hips with collapse grades of minimal (1), moderate (2), and severe (3) degrees were designated as an ordinal variable and tested for associations. The Kruskal-Wallis test with Monte Carlo significance of 10000 random seeded samples was used for nonparametric variables and the one-way ANOVA test was used for parametric variables. Ordinal logistic regression was carried out firstly to identify independent factors which predicted increasing severity of fracture collapse and secondly to identify factors which predicted increased likelihood to walk well after rehabilitation. A *p* value of <0.05 was taken as statistically significant.

## 3. Results

### 3.1. Baseline Factors and Univariate Analysis

Patients who have died or were lost to follow-up before 3 months were excluded from analysis unless they were already documented to have moderate or severe degrees of fracture collapse. Out of the 433 total patients treated with a DHS, 354 patients had a radiological follow-up of at least 3 months and 7 additional patients had an earlier known moderate or severe collapse. In all, 361 patients fulfilled the analysis criteria.

Of the 361 patients, 319 (88.4%) had an X-ray taken after at least 6 months and 293 (81.2%) after at least 9 months. The mean radiological follow-up was 14.6 months. Definite survival or mortality data was recovered for 360 (99.7%) patients at the time of data analysis which was 2.4 to 7.6 years after surgery (see Table 1).

Out of the 361 patients entered for analysis, 234 had minimal collapse, 84 had moderate collapse, and 43 had severe collapse. Out of the 43 patients with severe collapse, 20 patients had complete collapse where the lag screw or blade was touching the barrel and with no possibility for further collapse. This subgroup was, however, deemed too small for further analysis under a separate rank.

Patients with increasing severity of collapse were significantly older (mean age for minimal collapse = 83, moderate collapse = 82.6, severe collapse = 86, and *p* = 0.042), more likely suffered from A2 fractures (minimal = 40.2%, moderate = 65.5%, severe = 67.4%, and *p* = 0.000), and more likely had suboptimal tip-apex distance of more than 25 mm (minimal = 0%, moderate = 6%, severe = 0%, and *p* = 0.002) and poor reduction (minimal = 0.9%, moderate = 7.1%, severe = 18.6%, and *p* = 0.000) (see Table 2).

A number of poor outcomes were significantly associated with the severity of collapse, including nonfunctional walking status after rehabilitation (minimal collapse = 20.5%, moderate = 25%, severe = 34.9%, and *p* = 0.016), inability to maintain premorbid walking function (minimal collapse = 34.2%, moderate = 33.3%, severe = 62.8%, and *p* = 0.028), and reoperations (minimal = 0.9%, moderate = 2.4%, severe = 11.6%, and *p* = 0.002).

A number of adverse radiological features or complications were more common in those with increasing severity of collapse, including lateral wall fractures (minimal = 2.1%, moderate = 21.4%, severe = 55.8%, and *p* = 0.000), implant migration in the femoral head (minimal = 0.4%, moderate = 4.8%, severe = 11.6%, and *p* = 0.001), hip joint cutouts (minimal = 0%, moderate = 2.4%, severe = 11.6%, and *p* = 0.000), and nonunions (minimal = 0%, moderate = 1.2%, severe = 16.3%, and *p* = 0.000).

**Figure 2:**
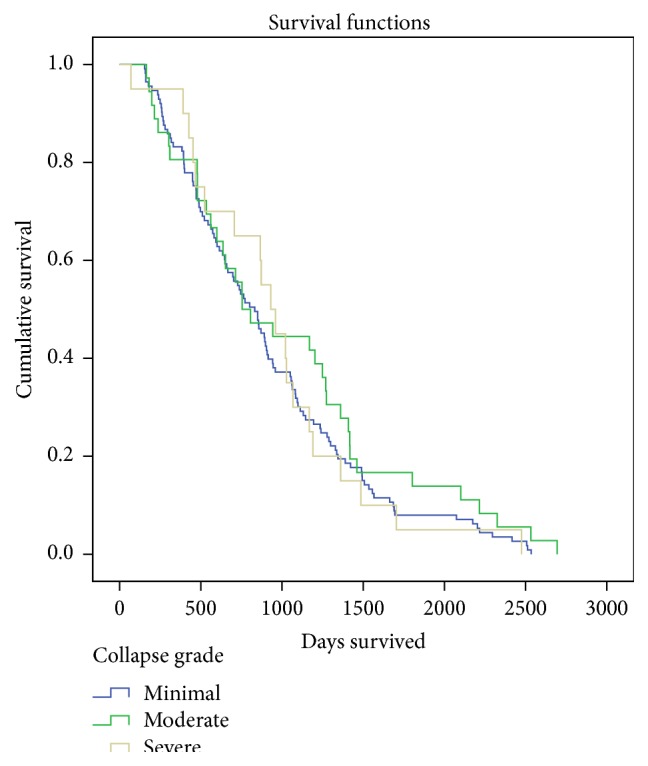
Kaplan-Meier survival plot of patients with different grades of fracture collapse up to 7.6 years after surgery; there was no statistically significant difference between patients with different group of collapse (log-rank test, *p* = 0.503).

The mean survival of patients with minimal collapse was 916 days (95% CI: 807–1025), with moderate collapse it was 1025 days (95% CI: 794–1256), and with severe collapse it was 944 days (95% CI: 721–1196). Patients with increasing severity of collapse had no difference in survival up to 7.6 years after surgery in Kaplan-Meier survival analysis (log-rank test, *p* = 0.503) (see Figure 2).

### 3.2. Multivariate Analysis

Based on ordinal regression, the independent risk factors for increased collapse were increasing age (*p* = 0.037), female sex (*p* = 0.024), 31.A2 fracture class (*p* = 0.010), increased operative duration (*p* = 0.011), poor reduction quality (*p* = 0.000), and suboptimal tip-apex distance of >25 mm (*p* = 0.050) (see Table 3).

Ordinal regression also showed that significant factors which independently predicted better functional waking status after operation were younger age (*p* = 0.036), higher MMSE marks (*p* = 0.000), higher MBI marks (*p* = 0.010), better premorbid walking status (*p* = 0.000), less severe fracture collapse (*p* = 0.011), and optimal lag screw position in centre-centre or centre-inferior position (*p* = 0.020) (see Table 4).

## 4. Discussion

In this study, it was demonstrated that fracture collapse was associated with poorer functional outcome, and this was independent of the patients' premorbid status. Moreover, complications occurred more commonly when patients had a more severe degree of collapse.

It is widely recognized in hip arthroplasty that shortening compromises the abductor muscle lever arm, resulting in weakness. In intracapsular neck of femur fractures, Zlowodzki et al. [12] studied 660 patients who have undergone screw fixation and concluded that a shortening of more than 10 mm resulted in significantly poorer short form 36 physical functioning scores compared to those with then 5 mm of shortening. Our study also suggests the same phenomenon to be true in geriatric intertrochanteric fractures, where increased fracture collapse indeed led to poorer walking function.

Our results showed that the independent risk factors for increased fracture collapse were old age, female sex, fracture comminution (31.A2 grading), increased operative time, poor fracture reduction quality, and suboptimal TAD. Of these factors, good fracture reduction appears to be of paramount importance. This is logical because doing so also effectively restores maximal contact of any available bony buttress [15, 21, 22]. For a dynamic device to work without excessive collapse, a majority of bone along the femur's circumference should remain intact and in contact. The well-known regions which provide effective bony support and load transfer between the main proximal and shaft fragments include the posterior medial calcar [23], the lateral wall [24], and the anteromedial region [25, 26]. As such, severe angulation, medialization of the proximal fragment [27], and lateral wall fractures [28, 29] result in a loss of bony support, and excessive fracture collapse.

Older female patients are more likely to have poor bone quality and reduced resistance to fracture collapse. A2 fractures are more comminuted and less stable than A1 fractures. As shown here, these patients were more likely to have eventual collapse. Fractures that required increased operative time also had increased likelihood of collapse. However, we are unable to conclude on whether it is prolonged operation itself or difficult fracture patterns which indirectly led to increased fracture collapse.

A number of studies have already pointed out the importance of implant position on outcomes. This was not consistency reproduced in our results possibly because of the low occurrences of poor TAD and poor implant positioning. Good implant positioning is known to be best determined by a small tip-apex distance of less than 25 mm and centre-centre or centre-inferior positioning of the lag screw in the femoral head in the AP and lateral radiographs [19, 22, 30, 31]. In all, surgeons must be meticulous with reduction and implant placement in trochanteric hip fractures while being mindful of risk factors such as osteoporosis and fracture comminution. The patients' functional outcome may be improved if precautions can be taken to limit the severity of fracture collapse.

There are a number of limitations to the study. Two slightly different implants were used in the group of patients, namely, as spiral blade and conventional lag screw. A standardized scoring system [32] was not used to grade functional outcomes because of the partially retrospective nature of data collection. We were not able to use an exact time point to define the patients' functional outcome as many of them suffered concomitant medical illness during or after rehabilitation and our study population had a highly variable timeline in recovery. We were unable to take pain into account in the clinical outcome analysis as we felt that there was poor documentation of this data in records. We were unable to find any verified grading system for the amount of collapse after DHS; nonetheless we felt that current method deemed simple enough and clinically applicable. The method through which we measured collapse of the metal implant may not always accurately reflect the true shortening between the bony fragments as an underestimation is likely in patients with implant migration in the femoral head.

The literature already contains many answered questions concerning improving patient survival and reducing complications. More research is needed to study how geriatric hip fracture patients can maintain better function. It is now evident that restoration and maintenance of the leg length is functionally important for both intracapsular [12, 13] and trochanteric hip fractures. Future implant design and improvements in treatment principles should take such findings into consideration.

## 5. Conclusion

Femoral shortening and collapse after DHS fixation are predisposed by old age, female sex, fracture comminution, poor reduction, and suboptimal implant placement. While the DHS is designed to allow for sliding and some degree of fracture collapse, those with severe collapse are more prone to eventual mechanical failure and complications. Increased collapse adversely affected patients' function in walking but did not appear to impair their survival.

## Figures and Tables

**Table 1:** Patient selection and follow-up characteristics.

Patients and follow-up characteristics		
Total patients	%	*n* = 433
Known moderate or severe collapse before 3 months	1.6%	7
Adequate follow-up at 3 months	81.8%	354

Fulfilled analysis criteria	83.4%	361

Survival data available 2.4–7.6 years post-op	99.7%	360
Had X-ray after 6 months	88.4%	319
Had X-ray after 9 months	81.2%	293

**Table 2:** Differences in baseline and outcome variables in relation to increasing severity of collapse.

Baseline and surgical variables							
Amount of collapse	Minimal		Moderate		Severe		*p* value
	%/mean	*n* = 234	%/mean	*n* = 84	%/mean	*n* = 43	
Age at operation	83	(SD = 0.75)	82.6	(SD = 0.85)	86	(SD = 0.62)	**0.042** ^**∗**^
Female versus males	59.8%	140	71.4%	60	74.4%	32	0.057
31.A2 versus A1	40.2%	94	65.5%	55	67.4%	29	**0.000**
Screw versus blade fixation	54.7%	128	58.3%	49	46.5%	20	0.461
Premorbid nonfunctional walker	6.8%	16	9.5%	8	2.3%	1	0.531
Premorbid assisted walker	10.7%	25	13.1%	11	14.0%	6	
Premorbid independent walker	81.2%	190	77.4%	65	83.7%	36	
Operation delayed more than 2 days after admission	10.3%	24	6.0%	5	2.3%	1	0.143
MMSE score	15.9	(SD = 7.4)	16.1	(SD = 7.7)	15.9	(SD = 7.4)	0.984^**∗**^
Modified Barthel Index	85.3	(SD = 22.4)	84.4	(SD = 25.1)	83.4	(SD = 23.9)	0.890^**∗**^
ASA score 3 or above	62.0%	145	63.1%	53	58.1%	25	0.862
Operated by trainees (<6 years of experience)	31.6%	74	29.8%	25	30.2%	13	0.941
Operative time	41.5 mins	(SD = 16.3)	43.6 mins	(SD = 18.6)	47.8 mins	(SD = 21.3)	0.084^**∗**^
Suboptimal centre-centre or centre-inferior lag screw position	27.4%	64	42.9%	36	39.5%	17	**0.019**
Suboptimal tip-apex distance > 25 mm	0.0%	0	6.0%	5	0.0%	0	**0.002**
Poor reduction (>4 mm translation and 5 degrees varus)	0.9%	2	7.1%	6	18.6%	8	**0.000**
Acceptable reduction (>4 mm translation or 5 degrees varus)	19.7%	46	42.9%	36	30.2%	13	
Good reduction (<4 mm translation and <5 degrees varus)	79.5%	186	50.0%	42	51.2%	22	
Perfect reduction (<2 mm translation and no varus)	59.4%	139	35.7%	30	30.2%	13	**0.000**

Outcomes							

Walking function							
Nonfunctional walker	20.5%	48	25.0%	21	34.9%	15	**0.016**
Assisted walker	26.9%	63	22.6%	19	34.9%	15	
Independent walker	52.6%	123	52.4%	44	27.9%	12	
Unable to maintain walking function	34.2%	80	33.3%	28	62.8%	27	**0.028**

Cumulative mortality							
Died at 6 months	2.1%	5	1.2%	1	2.3%	1	0.880
Died at 1 year	8.5%	20	8.3%	7	2.3%	1	0.377
Died at 2 years	22.2%	52	19.0%	16	16.3%	7	0.628

Complications							
Lateral wall fractures	2.1%	5	21.4%	18	55.8%	24	**0.000**
Any mechanical failure	0.4%	1	7.1%	6	23.3%	10	**0.000**
Implant migration in femoral head	0.4%	1	4.8%	4	11.6%	5	**0.001**
Hip joint penetration and cutout	0.0%	0	2.4%	2	11.6%	5	**0.000**
Side plate pullout	0.0%	0	0.0%	0	2.3%	1	0.111
Nonunion	0.0%	0	1.2%	1	16.3%	7	**0.000**
Infection	0.4%	1	0.0%	0	4.7%	2	0.053
Reoperations	0.9%	2	2.4%	2	11.6%	5	**0.002**

Kruskal-Wallis test with Monte Carlo significance for nonparametric variables.

^*∗*^One-way ANOVA test for continuous variables.

**Table 3:** Ordinal regression of factors which predicted increasing severity of collapse. Value with a positive (+) estimate predicts more fracture collapse and that with a negative (−) estimate predicts less.

Estimated likelihood of increased fracture collapse in ordinal regression							
	Estimate	Standard error	Wald	df	Sig.	95% confidence interval	
Per day delay from admission to operation	−0.121	0.191	0.401	1.000	0.527	−0.496	0.254
Operative time per minute increase	0.021	0.008	6.484	1.000	**0.011**	**0.005**	**0.037**
Age at operation per year increase	0.049	0.023	4.369	1.000	**0.037**	**0.003**	**0.095**
MMSE per mark increase	0.025	0.023	1.182	1.000	0.277	−0.020	0.071
MBI per mark increase	0.007	0.008	0.701	1.000	0.402	−0.009	0.023
Poor premorbid walking status (independent versus assisted versus dependent)	−0.278	0.282	0.974	1.000	0.324	−0.831	0.274
Poor reduction quality (good versus acceptable versus poor)	1.112	0.240	21.510	1.000	**0.000**	**0.642**	**1.582**
Male versus female	−0.680	0.302	5.067	1.000	**0.024**	**−1.271**	**−0.088**
31.A1 class versus A2	−0.719	0.281	6.570	1.000	**0.010**	**−1.269**	**−0.169**
Screw versus blade	−0.156	0.284	0.301	1.000	0.583	−0.712	0.401
Operated by specialists (>6 years of experience)	0.360	0.311	1.338	1.000	0.247	−0.250	0.971
Suboptimal centre-centre or centre-inferior lag screw position	0.128	0.295	0.190	1.000	0.663	−0.449	0.706
ASA 1-2 versus 3-4	0.194	0.292	0.441	1.000	0.506	−0.378	0.765
Suboptimal tip-apex distance > 25 mm	1.978	1.011	3.829	1.000	**0.050**	**−0.003**	**3.959**

Pseudo *R*-square (Nagelkerke) = 0.244							

**Table 4:** Ordinal regression of factors which predicted better functional walking status after rehabilitation. Value with a positive (+) estimate predicts better walking status and that with a negative (−) estimate predicts a worse outcome.

Estimated likelihood of having better walking function in ordinal regression							
	Estimate	Standard error	Wald	df	Sig.	95% confidence interval	
Per day delay from admission to operation	0.069	0.194	0.126	1.000	0.722	−0.311	0.448
Operative time per minute increase	0.003	0.009	0.085	1.000	0.771	−0.014	0.020
Age at operation per year increase	−0.051	0.024	4.421	1.000	**0.036**	**−0.099**	**−0.003**
MMSE per mark increase	0.086	0.023	13.344	1.000	**0.000**	**0.040**	**0.132**
MBI per mark increase	0.021	0.008	6.587	1.000	**0.010**	**0.005**	**0.037**
Poor premorbid walking status (independent versus assisted versus dependent)	1.665	0.323	26.565	1.000	**0.000**	**1.032**	**2.297**
Poor reduction quality (good versus acceptable versus poor)	0.331	0.275	1.444	1.000	0.230	−0.209	0.871
Collapse grade (minimal versus moderate versus severe)	−0.650	0.256	6.445	1.000	**0.011**	**−1.151**	**−0.148**
Male versus female	−0.213	0.292	0.530	1.000	0.467	−0.786	0.360
31.A1 class versus A2	0.072	0.291	0.061	1.000	0.804	−0.498	0.642
Screw versus blade	−0.105	0.298	0.124	1.000	0.724	−0.690	0.480
Operated by specialists (>6 years of experience)	0.090	0.321	0.078	1.000	0.780	−0.540	0.719
ASA 1-2 versus 3-4	0.280	0.306	0.840	1.000	0.359	−0.319	0.880
No mechanical failure	0.787	0.684	1.326	1.000	0.250	−0.553	2.127
Not reoperated	1.779	1.046	2.890	1.000	0.089	−0.272	3.830
No lateral wall fracture	−0.476	0.526	0.820	1.000	0.365	−1.506	0.554
Suboptimal tip-apex distance > 25 mm^*∗*^	22.688	0.000	—	1.000	—	22.688	22.688
Suboptimal centre-centre or centre-inferior lag screw position	−0.695	0.299	5.392	1.000	**0.020**	**−1.282**	**−0.108**

Pseudo *R*-square (Nagelkerke) = 0.504							

^*∗*^Not enough valid cases to compute the significance of this item.
